# The genomic structure of a human chromosome 22 nucleolar organizer region determined by TAR cloning

**DOI:** 10.1038/s41598-021-82565-x

**Published:** 2021-02-04

**Authors:** Jung-Hyun Kim, Vladimir N. Noskov, Aleksey Y. Ogurtsov, Ramaiah Nagaraja, Nikolai Petrov, Mikhail Liskovykh, Brian P. Walenz, Hee-Sheung Lee, Natalay Kouprina, Adam M. Phillippy, Svetlana A. Shabalina, David Schlessinger, Vladimir Larionov

**Affiliations:** 1 Developmental Therapeutics Branch, National Cancer Institute, Bethesda, MD 20892 USA; 2 National Center for Biotechnology Information, National Library of Medicine, Bethesda, MD 20892 USA; 3 Laboratory of Genetics and Genomics, National Institute on Aging, Baltimore, MD 21224 USA; 4 Computational and Statistical Genomics Branch, National Human Genome Research Institute, Bethesda, MD 20892 USA

**Keywords:** Genetics, Molecular biology, Genomics

## Abstract

The rDNA clusters and flanking sequences on human chromosomes 13, 14, 15, 21 and 22 represent large gaps in the current genomic assembly. The organization and the degree of divergence of the human rDNA units within an individual nucleolar organizer region (NOR) are only partially known. To address this lacuna, we previously applied transformation-associated recombination (TAR) cloning to isolate individual rDNA units from chromosome 21. That approach revealed an unexpectedly high level of heterogeneity in human rDNA, raising the possibility of corresponding variations in ribosome dynamics. We have now applied the same strategy to analyze an entire rDNA array end-to-end from a copy of chromosome 22. Sequencing of TAR isolates provided the entire NOR sequence, including proximal and distal junctions that may be involved in nucleolar function. Comparison of the newly sequenced rDNAs to reference sequence for chromosomes 22 and 21 revealed variants that are shared in human rDNA in individuals from different ethnic groups, many of them at high frequency. Analysis infers comparable intra- and inter-individual divergence of rDNA units on the same and different chromosomes, supporting the concerted evolution of rDNA units. The results provide a route to investigate further the role of rDNA variation in nucleolar formation and in the empirical associations of nucleoli with pathology.

## Introduction

To sustain the growth of any cell, about half of all RNA synthesis is ribosomal RNA, and variable rates of transcription are maintained in part by the existence of multiple copies of rDNA in the cell nucleus. In humans, approximately 300 rDNA repeats are distributed among five nucleolar organizer regions (NORs) on the short arms of the acrocentric chromosomes 13, 14, 15, 21 and 22^1,2^.

**Figure 1:**
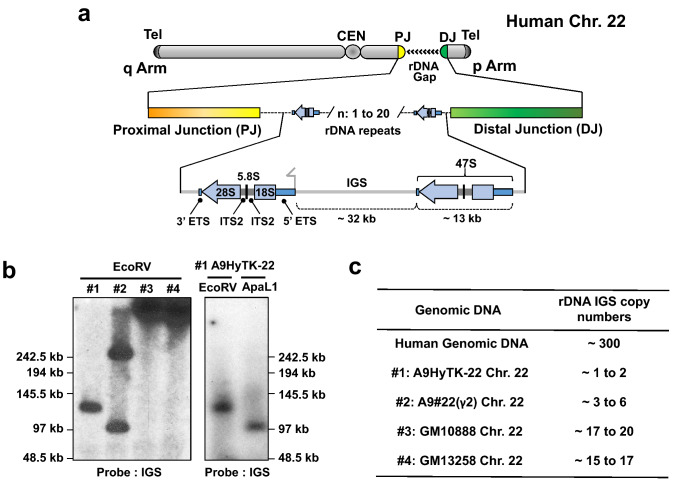
Schematic representation of the acrocentric human chromosome 22. (**A**) Scheme of the chromosome 22 with the rDNA portion and proximal (PJ) and distal (DJ) junction sequences. The number of rDNA units (n) on chromosome 22 varies from 1 to 20. The ~ 13 kb transcribed human 47S rDNA region shown as a blue arrow. Each rDNA unit is composed of the transcribed region encoding 47S rRNA (5′ETS, 18S, ITS1, 5.8S, ITS2, 28S and 3′ETS) and a ∼32 kb IGS (intergenic spacer sequence). (**B**) Southern blot analysis of four mouse/human hybrid cell lines containing human chromosome 22. After EcoRV digestion of DNA isolated from each hybrid cell line, the rDNA repeats were detected with a probe specific to the rDNA intergenic spacer (IGS). Southern blot analysis of the A9HyTk-22 mouse/human cell line was also performed after ApaLI endonuclease digestion. (**C**) A copy number of the rDNA repeats in each hybrid cell line was estimated by quantitative real-time PCR (qPCR) with a set of the primers specific to the IGS.

The number of rDNA repeats in individual human NORs ranges from 1–3 to more than 140 copies^2–4^, and only 20–50% of all RNA genes are transcriptionally active in human cells at any time^5^. Each repeat contains a ~ 13 kb pre-rRNA coding sequence containing a copy of 18S, 5.8S, and 28S rRNA sequence separated by spacers, followed by a ~ 32 kb intergenic spacer (IGS) (Fig. 1A). The large precursor transcript (47S pre-rRNA) is synthesized by polymerase I and then processed into the mature rRNA species^6^.

The structure of a 43 kb human ribosomal DNA unit was first defined in 1995, based on the sequence analysis of dozens of rDNA-containing sequences available from plasmid and cosmid libraries^7^. These clones were highly homologous both in their coding region and IGS^8^. However, decades later, only a few rDNA-containing BAC clones have been sequenced from individuals. Consequently, the full extent of variation in single rDNA repeats as well as the genomic structure of rDNA clusters have remained unknown, representing gaps totaling ~ 0.5% in the current genomic assembly.

Systematic studies of sequences that lie proximal (centromeric) and distal (telomeric) to rDNA arrays (named proximal and distal junction sequences, PJ and DJ) have been carried out^9,10^. Floutsakou et al. identified these sequences for rDNA arrays on acrocentric chromosomes, based on sequences of BAC and cosmid clones available in GenBank^9^. Comparison of these sequences with DJ contigs derived from acrocentric chromosomes propagated in monochromosomal somatic cell hybrids revealed that DJ’s are nearly identical among all five pairs of acrocentric chromosomes^10^. Such conservation is consistent with frequent recombination between the rDNA arrays on the acrocentric chromosomes^8,11^ and is consistent with a possible function of DJ in nucleolar organization. In fact, van Sluis et al.^10^ showed that all DJ regions encode transcripts that, when depleted, result in nucleolar stress. By contrast, PJ sequences remain poorly characterized. PJ sequences are almost entirely segmentally duplicated, which makes their assembly difficult. However, this does not rule out that PJ may also contain elements that regulate NOR function. Given the inferred involvement of NORs not only in ribosome biogenesis but also in processes as varied as regulation of mitosis, cell-cycle progression and proliferation, many forms of stress response and biogenesis of multiple ribonucleoprotein particles in diseases and aging [reviewed by McStay^4^], we have aimed to characterize the structure of the entire rDNA array in an individual human NOR. Most of the 47S variants were also found in independently sequenced cellular rRNA, including a number that are consistently present at allele frequencies up to ~ 50%. The variants thus revealed significant heterogeneity in human rDNA, raising the possibility that variants may modulate ribosome formation or function.

In this work, a modified TAR cloning strategy has been applied to characterize the entire NOR region on a human chromosome 22 that harbors a relatively low number of rDNA repeats^3,12,13^, facilitating the analyses. The first entire chromosomal span of a human rDNA array, containing two rDNA units along with PJ and DJ, has thereby been cloned, sequenced, and analyzed.

## Results

### Strategy for isolating rDNA sequences from the human chromosome 22

The entire NOR sequence from chromosome 22 was isolated using transformation-associated recombination (TAR) cloning procedures^14^. This technique is based on in vivo recombination between genomic DNA and a TAR vector containing 5′ and 3′ short targeting sequences common to the target DNA and allows selective isolation of any specific genomic region from total genomic DNA. Previously we demonstrated that treatment with a cutting restriction enzyme(s) or Cas9 nucleases to create specific double strand breaks bracketing the target genomic DNA sequence increases the yield of region-positive clones approximately 20–30 times^15^. But to do it effectively, we need to know exact DNA sequence information of the cloning region.

In this work, to avoid cloning of rDNA from more than one acrocentric chromosome, we started from genomic DNA isolated from mouse-human hybrid cell lines that contain only a single human chromosome 22. For analysis, we chose four cell lines, A9HyTK-22^13^, A9#22(γ2)^16^, GM10888^17^, and GM13258^18^.

Before the cloning step, we analyzed the size of rDNA clusters in each cell line by Southern blot hybridization (Fig. 1B). For this purpose, genomic DNA from each hybrid cell line was digested by EcoRV endonuclease. This nuclease has no (or very rare) recognition sites in human rDNA units but these sites are present in the junction sequences^12^. EcoRV-digested genomic DNA was separated by CHEF (Contour-Clamped Homogeneous Electric Field) and hybridized with a probe specific to the human rDNA spacer sequence (see “Materials and methods” for details and Supplementary Table S1). A single band approximately 130 kb in size was observed for the A9HyTK-22 cell line (Fig. 1B; #1), suggesting a content of more than one unit but less than three units of rDNA. Genomic DNA from A9HyTK-22 cells was also digested with ApaLI endonuclease, because preliminary examination of available rDNA sequences showed that recognition sites for ApaLI are also not common in human rDNA units but are present in the junction sequences. ApaLI digest resulted in one band approximately 110 kb in size (Fig. 1B; #1), further supporting our prediction of rDNA units on the chromosome 22 in this A9HyTK-22 cell line.

We also assessed the number of copies of human rDNA in A9HyTK-22, A9#22(γ2), GM10888, and GM13258 cell lines by qPCR analysis, using primers specific for human spacer sequence (Supplementary Table S1). Consistent with the analysis of restriction digests, based on qPCR, A9HyTK-22 and A9#22(γ2) cell lines were inferred to contain 1 to 2 and 3 to 6 human rDNA repeats, respectively; and GM10888 and GM13258 cell lines, approximately 18 and 16 units, respectively (Fig. 1C). For further TAR cloning experiments, we chose the A9HyTK-22 cell line, containing the smallest number of rDNA units.

### TAR isolation of the rDNA gene cluster sequence from the chromosome 22

If the rDNA cluster on the chromosome 22 lacks both EcoRV and ApaLI restriction recognition sites (Fig. 1B), the entire block of rDNA cluster could be TAR-isolated as an EcoRV or ApaLI fragment.

**Figure 2:**
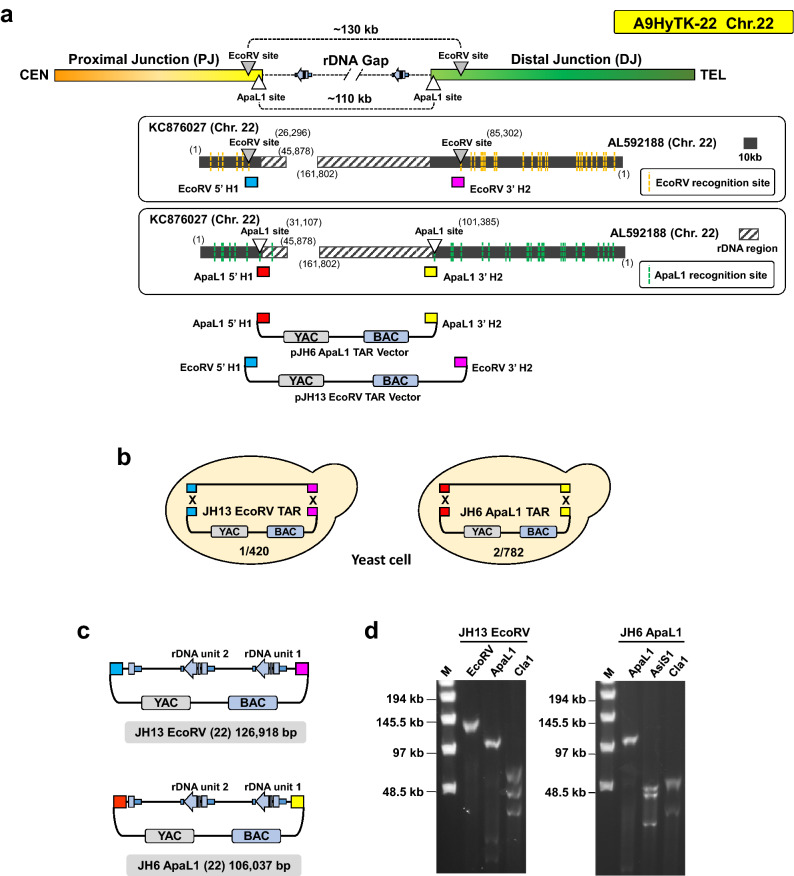
Designing of TAR cloning vector and Closing the rDNA gap on chromosome 22. (**A**) Schematic diagram illustrating the expected innermost EcoRV (gray triangle) or ApaL1 (white triangle) enzyme recognition sites of the human chromosome 22 rDNA gap region on A9HyTk-22 Mouse/Human monochromosomal hybrid cell line. The lower panels show EcoRV (yellow dotted lines) or ApaL1 (green dotted lines) recognition sites on human chromosome 22 proximal junction (PJ) cosmid (KC876027) and distal junction (DJ) BACs (AL592188). The rDNA repeats are indicated by black stripes pattern box. Two TAR vectors, pJH6 and pJH13, containing YAC and BAC cassettes and unique hooks were constructed. pJH6 vector contains ApaLI-5′hook1 (in red) and ApaLI-3′hook2 (in yellow). Vector pJH13 contains EcoRV-5′hook1 (in blue) and EcoRV-3′hook2 (in pink). EcoRV-5′hook1 is located 6,249 bp upstream the rDNA cluster. EcoRV-3′hook2 is located 16,278 bp downstream the rDNA cluster. (**B**) The diagrams show TAR cloning of the entire rDNA cluster on the chromosome 22 from total genomic DNA isolated from the A9HyTK-22 hybrid cell line and digested either by EcoRV or ApaLI with two linearized TAR vectors. Homologous recombination between the targeting sequences in the vectors and the targeted sequences on the chromosome 22 leads to the rescue of the rDNA cluster as circular TAR/YAC/BAC molecules, i.e. JH13 EcoRV and JH6 ApaLI. Using the pJH13 EcoRV vector, 1 out of 420 screened colonies were positive and the pJH6 ApaL1 vector was 2/782, respectively. (**C**) TAR isolates were transferred from yeast to *E. coli* cells for BAC DNA isolation and further sequencing. The length of the insert in the JH13/EcoRV-BAC is 126,918 bp. The length of the insert in the JH6/ApaLI-BAC is 106,037 bp. (**D**) The size of the rDNA cluster after BACs linearization by either EcoRV or ApaLI. M—a size marker [CHEF DNA Size Lambda Ladder (BIO-RAD)].

Based on our earlier demonstration that double strand breaks near the targeted sequences make them highly recombinogenic^15^, we designed two vectors, pJH13 and pJH6, to TAR-clone either the EcoRV-digested 130 kb or the ApaLI-digested 110 kb chromosome 22 rDNA cluster from the A9HyTK-22 hybrid cells (see “Materials and methods” for details). The targeting hooks were chosen from the PJ sequence corresponding to a cosmid sequence KC876027^9^ and DJ sequence corresponding to a distal junction (DJ) BAC (AL592188) including other common to all acrocentric chromosomes^10^ (Fig. 2A).

To prove that positions of the chosen hooks are correctly chosen, we verified the positions of ApaLI and EcoRV sites using a modified Cas9-Assisted Targeting Chromosome segments (CATCH) method^19^ (Supplementary Fig. 1A–D). Using this approach, > 200-fold enrichment for reads corresponding to the target region was achieved. Illumina sequencing of DNA reads corresponding to human rDNA clearly demonstrated that the DNA bands included rDNA repeats and flanking sequences of rDNA repeats that match/align with the sequences nearest to the first EcoRV and ApaLI recognition sites in the annotated PJ (KC876027) and DJ (AL592188) sequences (Supplementary Fig. 1E). This result confirmed that the chosen targeting hooks for DJ and PJ are suitable for cloning the entire rDNA array of the chromosome 22.

ApaLI-5′hook1 corresponds to 177 base pairs at position 31,083–31,259 in KC876027. ApaLI-3′hook2 corresponds to 179 base pairs at position 101,370–101,550 in the NOR distal junction of KC876027. The EcoRV-5′hook1 corresponds to 196 base pairs at position 26,273–26,468 in KC876027. EcoRV-3′hook2 corresponds to 199-base pairs at position 85,266–85,464 in the NOR distal junction of AL592188 sequence (Fig. 2A).

Before TAR cloning, TAR vectors were linearized by AscI/NotI (Supplementary Fig. S2) to expose the targeting sequences. Genomic DNA from the A9HyTK-22 hybrid cells was prepared, digested either by EcoRV or ApaLI endonuclease, and transformed into yeast spheroplasts along with a linearized TAR vector. Recombination between the targeting hooks in the vector and the targeted sequences on the chromosome 22 leads to the rescue of the target regions as circular TAR/YAC molecules (Fig. 2B). To identify TAR/YAC containing the predicted regions, His^+^ yeast transformants were combined into pools and checked by diagnostic primers (regions 14 and 16 in Supplementary Table S1; Supplementary Figs. S3A,B).

To check the fidelity of the TAR-cloned material after transfection into bacterial cells, several approaches were taken. First, the individual bacterial colonies were checked by PCR using diagnostic primers (regions 14 and 16 in Supplementary Table S1; Supplementary Fig. S3C). Then the size of the cloned material was checked by CHEF gel electrophoresis after BAC linearization by either EcoRV or ApaLI (Fig. 2D). To prove the presence of the rDNA sequence, DNAs isolated from 130 and 110 kb BACs were examined by PCR (Supplementary Table S1; Supplementary Fig. S3D). Further sequencing of JH13/EcoRV-BAC and JH6/ApaLI- BAC clones revealed 126,918 bp and 106,037 bp sequences that each contains two tandem rDNA units (Fig. 2C,D) and completely cover the rDNA on human chromosome 22 from this hybrid cell line. The annotated assemblies of the BAC sequences are available in GenBank (Supplementary Table S2A).

### Assembly of the entire NOR on the chromosome 22

Recently McStay and colleagues sequenced and assembled DJ DNA sequence contig that abuts rDNA arrays on the telomeric side of all five acrocentric chromosomes^10^. Their analysis revealed remarkable sequence and functional conservation among human acrocentric chromosomes. However, such analysis has not been carried out for the PJ DNA sequence and the sequence was remained as a gap on chromosome 22. Therefore, we first verified the PJ DNA sequence that abuts the rDNA cluster on the centromeric side of the chromosome 22 in the A9HyTK-22 cell line by TAR cloning.

**Figure 3:**
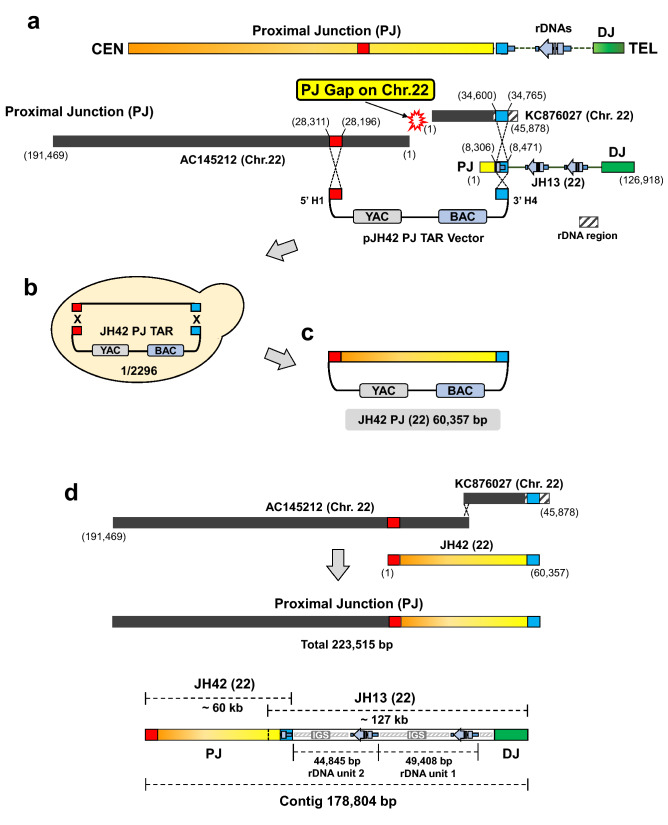
TAR isolation of the proximal junction sequence of the chromosome 22. (**A**) The pJH42 vector containing YAC and BAC cassettes was constructed to clone the sequences proximal to the rDNA cluster. The pJH42 vector contains two unique hooks, H1 (in red) and H4 (in blue). H1 sequence was designed from AC145212 BAC. H4 sequence was designed from cosmid KC876027/JH13 EcoRV (22) BAC. The rDNA region is indicated by black stripes pattern box on KC876027. (**B**) The diagram shows TAR cloning of the PJ sequence. Total genomic DNA isolated from the A9HyTK-22 cells along with a linearized TAR vector were co-transformed into yeast *Saccharomyces cerevisiae* cells. Homologous recombination between the H1 and H4 targeting sequences in the vector and the targeted sequences on the chromosome 22 leads to rescue of the region of interest as a circular TAR/YAC molecule (JH42-BAC). (**C**) TAR isolates were transferred from yeast to *E. coli* cells for BAC DNA isolation and further sequencing. The length of the insert in the JH42-BAC is 60,357 bp. (**D**) A 178,804 bp contig of the NOR on the chromosome 22 in the A9HyTK-22 cell line was assembled, based on the sequences of JH42-BAC (PJ) and JH13/EcoRV-BAC (rDNA cluster).

For this purpose, TAR vector pJH42 containing two unique hooks, H1 and H4, was constructed (Fig. 3A). As described above*,* recombination between the hooks in the vector and the targeted sequences on the chromosome 22 leads to the rescue of the target region as a circular TAR/YAC molecule (Fig. 3B,C).

JH42-BAC DNA was isolated and sequenced. 60,357 bp sequence of JH42-BAC verifies PJ DNA sequence that abuts the rDNA cluster on the centromeric side of chromosome 22 in the A9HyTK-22 cell line. The annotated assembly of the BAC sequence is available in GenBank (Supplementary Table S2A).

**Figure 4:**
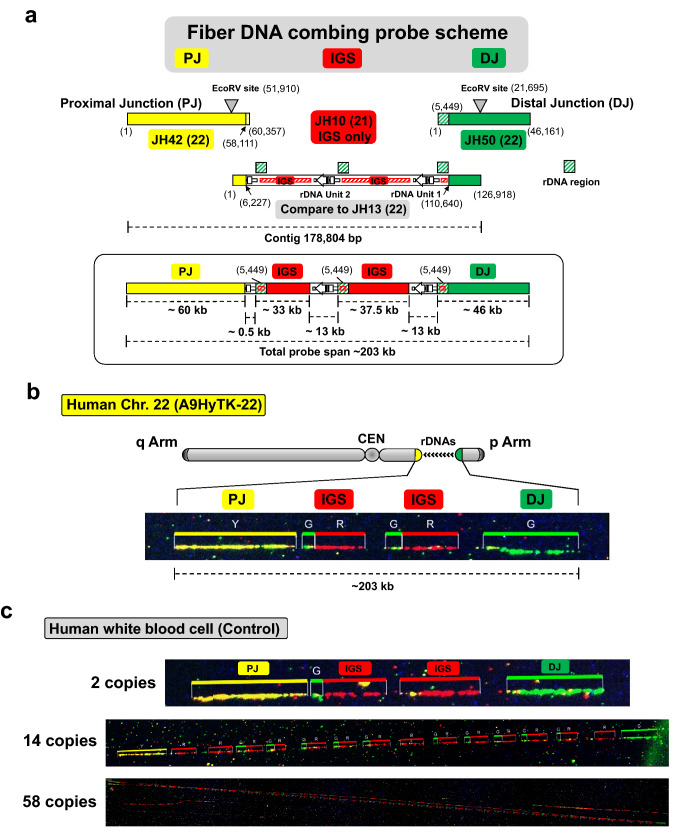
Confirmation of the NOR chromosome 22 contig by three-color DNA combing. (**A**) Scheme of the three-color DNA combing probe signal locations for the analysis of the rDNA cluster with the flanking PJ and DJ regions in A9HyTK-22 Chr. 22: JH42-BAC for PJ (in yellow), JH50-BAC for DJ (in green) and JH10-BAC (accession MF164270)^20^ for IGS (in red). (**B**) Representative DNA fiber segment. Single DNA fibers from hybrid A9HyTK-22 Chr. 22 cells that contain two rDNA units were stretched on CombiCoverslips (Genomic Vision). Color-bars show the signal distributions on DNA corresponding to the chromosome 22 NOR region. The order of the probes was detected by DNA combing using three probes: JH42-BAC DNA for PJ (in yellow), JH50-BAC for DJ (in green) and JH10-BAC DNA for IGS (in red). Signal distribution follows the proposed contig assembly. Note that JH50-BAC contains approximately 5.4 kb of the rDNA unit corresponding to the rDNA promoter region. Therefore, the small green signals are also seen in the cluster of the rDNA units. (**C**) Representative DNA fiber segments from white blood cells. The ~ 5.4 kb green signals are also seen in the cluster of the rDNA units.

Thus, based on the sequences of JH42-BAC (PJ), JH13/EcoRV-BAC (rDNA cluster) and JH6/ApaLI-BAC (rDNA cluster), a 178,804 bp contig of the NOR on the chromosome 22 in the A9HyTK-22 cell line was assembled (Fig. 3D). To confirm the overall structure of the assembled contig on the chromosome 22 in the A9HyTK-22 hybrid cell line, we performed molecular DNA combing analysis and fluorescence in situ hybridization (FISH). To generate three distinctive signals for the NOR region, including PJ, DJ, and IGS (rDNA), we used JH42-BAC for PJ, JH10-BAC^20^ for IGS only and JH50-BAC for DJ region (Fig. 4A, Supplementary Fig. S4). These distinctive probes thus do not detect the 13 kb rDNA gene region (Fig. 4A).

For molecular DNA combing analysis, DNA fibers were prepared from A9HyTK-22 cells. As seen in Fig. 4B, the expected ~ 203 kb NOR structure along with PJ and DJ on the human chromosome 22 was observed. As a control, human white blood cells were used. Interestingly, we also observed same 2 copies of rDNA repeat pattern on the white blood cell DNA fibers similar to the A9HyTK-22 cell line. In the white blood cells, NOR size range ~ 90 kb to ~ 2.6 Mb with corresponding numbers from 2 to 58 copies of rDNA repeats were observed (Fig. 4C). FISH analysis showed that the signals of all three probes are closely located on the human NOR regions in the A9HyTK-22 and human RPE cells (Supplementary Fig. S5).

### Comparative analysis of variants in rDNA units on human chromosomes 22 and 21

In this study, the comparison of the chromosome 22 rDNA units with each other and comparative analysis of rDNA units from the chromosome 22 and chromosome 21 were carried out separately. We compared the large-scale structure of the human chromosome 22 nucleolar organizer region with two rDNA units (MT497459; Supplementary Table S2) to GL000220.1 (AL592188) and KI270733, the UCSC and GenBank sequences that has historically served as a chromosome 22 rDNA reference, and which also contain two copies of rDNA unit flanked by ~ 90 kb sequence corresponding to DJ and an incomplete rDNA unit. The diversity of the complete rDNA units of the chromosome 22 in individuals (both same and different) was analyzed using Owen and Clustal software for pairwise^21,22^ and for multiple alignments, respectively. We found that the levels of intra- and inter-individual diversity in complete rDNA units located on the chromosome 22 are comparable (Supplementary Table S3 and S4).

For comparison of rDNA units located on chromosomes 22 and 21, we used chromosome 22 complete rDNA units (from this study and from UCSC and GenBank) and earlier identified complete rDNA copies of the chromosome 21 of different individuals (14 different rDNA units) as well as their suitable reference sequence from our previous publication^20^ (GenBank accession KY962518). For these chromosomes (22 and 21), comparable levels of intra- and inter-individual diversity in complete rDNA units were found (Supplementary Tables S4). In detail, the distribution of rDNA divergence in pairwise comparisons of completely sequenced units is shown in Figure S6 with an average value of 130 (± 7.3). The comparisons show that between half and two thirds of the variants (see Supplementary Table S4, shown in red and yellow) are common/universal between chromosomes 22 and 21 in different individuals. A majority of the common/universal variants discovered here were also recovered or validated from independent WGS or RNAseq data^20^, indicating that they are true variants and not the result of sequencing or cloning errors (see Supplementary Table S4 and “Discussion”). Whole-genome validation experiments were based on four samples with available high-coverage Illumina and PacBio data (see “Materials and methods”). To assess the extent of expression of the identified 45S variants, we examined the available RNA-seq datasets including one from the AK1 cell line and two, pre-rRNA-enriched, nucleolar datasets from the K562 cell line (see Supplementary Methods for details).

Based on the overall frequencies of alleles in RNAseq data, it is likely that variants are randomly distributed in repeats on the five human acrocentric chromosome pairs. In detail, the distribution of rDNA divergence in pairwise comparisons of completely sequenced units is shown in Supplementary Fig. S6 with an average value of 130 (± 7.3). We estimated an average of 122 ± 5.8 and 131.6 ± 6.8 variants per pairwise alignment of chromosome 22 and chromosome 21 rDNA units for different individuals. Thus, our data are comparable with the intra-individual variation in rDNA from the DNAseq data of a Utah CEPH-1463 trio at an intra-individual variant allele frequency (iVAF) of greater than ~ 5%^23,24^. An average of 192.7(± 14.3) and 85.3 (± 4.2) variants per individual at an iVAF of greater than 2% and 10% accordingly was also shown in Babaian’s study^23^. Our findings are thus in complete agreement with the previously suggested mechanism of concerted evolution of rDNA units.

### Distribution of variants in spacer regions and mature rRNA

The distribution of variants is not uniform across different functional regions of rDNA. The total numbers of variants in IGS is about twice as high as in the transcribed region. Of those in mature rRNA, by far the highest density is in 28S rRNA (~ 4.1–5 variants per kb); very few variants are observed in 18S, and none in 5.8S rRNA.

We analyzed RNA folding differences for 189 sequence variants, including 54 variations mapped to the transcribed region of chromosome 22 rDNA units. Most variants are located in expansion segments (ESs), which are regions that diverge relatively more during evolution, defined by comparing the rRNA structures of different species. In 28S rRNA, ES are enriched for both universal and rare/unique variants including SNPs and indels, whereas both types of variants are uncommon for the core regions of rRNAs. The location and distribution of the identified 28S rRNA variants in ES is comparable for chromosomes 21 and 22. All 13 variants from chromosome 22 unit 1 and 15 of 17 variants from chromosome 22 unit 2 were previously detected in 28S rRNAs when analyzing 13 rDNA units from chromosome 21^20^. All of these universal variants were independently found using WGS, and a number were confirmed as transcribed into rRNA by RNAseq data (Supplementary Table S4). Our study also supported and defined as universal 8 of 9 high frequency variants in 28S rRNAs that are prevalent in the population and abundant within the individual (population variant-detection frequency > 0.5, and an intra-individual variant allele frequency (iVAF) 0.33–0.66^23^.

The same two hot spots of variation (indels) in 28S rRNAs are located on the opposite strands of hairpin structures of ES27 and ES15 in chromosomes 22 and 21. Unlike variants elsewhere in the rRNA, these INDELs jointly occur frequently in both spots; and they are neighbors in RNA folding despite separation by ∼175–180 and 22–30 nucleotides in the primary sequences of ES27 and ES15 hairpins accordingly (Supplementary Figs. S7 and S8).

These results agree with recent evidence of concerted conformational dynamics and compensatory evolution of expansion segments to maintain features of secondary structure. The presence of individual variability of rDNA units in the same and different chromosomes in ES supports the idea that mature rRNAs may have specific sequences and structural variability adapted for specialized functions in different conditions or in a tissue-specific manner^20,25,26^.

The variants in ES regions did not in general show any likely significant modulation of 2D RNA structures. By contrast, the situation is quite different for several outliers that are mapped to “most highly conserved” rather than ES segments were found in both 28S rRNAs (G59A) and 18S (U682C) and were further analyzed. The G59A single nucleotide polymorphism (SNP) in 28S rRNA shows strongly biallelic expression (K562 total RNAseq)^23,27^ and modulates RNA folding, increasing the possibility that it might create some functional heterogeneity of the large ribosomal subunit (LSU). Only a single variant that was not seen on chromosome 21 clones, U682C, and has not been previously observed, was found in 18S rRNAs on rDNA unit 1 of chromosome 22. This specific variant is predicted to be a potential alteration of RNA structure (riboSNitch) in 18S rRNA that can modulate RNA-RNA interactions (Supplementary Fig. S9) and has been validated by “BLAST” searches against rRNA databases^28,29^.

### Location of variants outside of mature rRNA species

**Figure 5:**
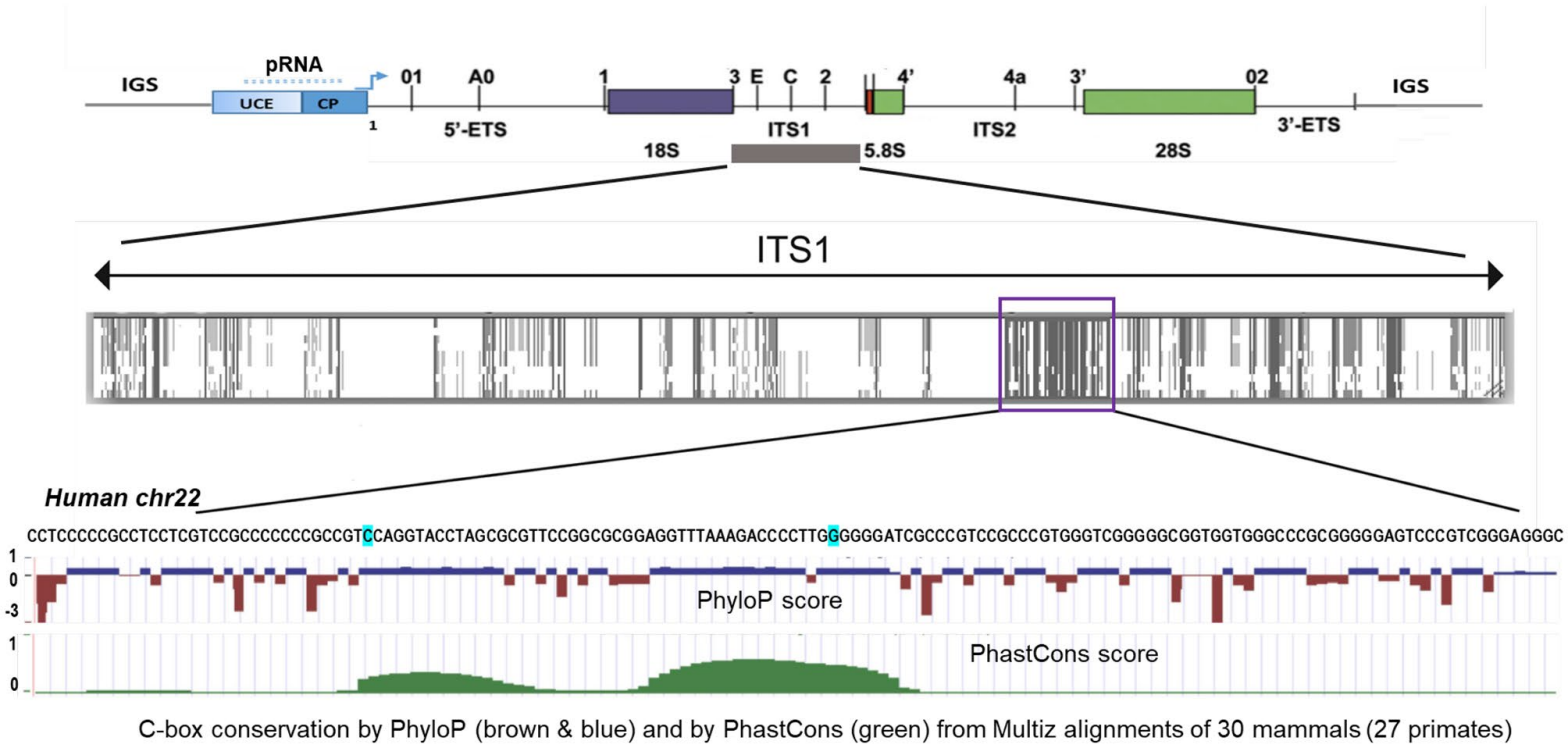
Human pre-rRNA processing and ITS1 and C-box similarity in mammals. Schematic presentation of the human RNA polymerase transcript—45Svpre-rRNA (upper panel), processing sites are marked as reviewed in Mullineux and Lafontaine^61^. UCE—upstream control element; CP—core promoter; IGS—an intergenic spacer; 5′ETS and 3′ETS—5′ and 3′ external transcribed spacers; ITS1 and ITS2—internal transcribed spacers 1 and 2. Conservation in mammals is indicated by shades of grey (a middle panel); C-box is shown in violet box. The C-box nucleotide sequence is shown for human chromosome 22 (this study; bottom panel); the locations of human variants (INDELs) in the C-box are highlighted in blue. C-box conservation by Phylop (brown and blue) and by PhastCons (green) from the Multiz alignment of 30 mammals is shown in the bottom panel (UCSC genome browser—https://genome.ucsc.edu/cgi-bin/hgGateway?hgsid=970436711_JhFQiQl4MIataJe6MUxuOqVnPRWz).

How might 47S region variations influence ribosome biogenesis and/or processing of the 47S pre-rRNA? The several ETS and ITS variants which are universal and found in both chromosomes as well as in the independent WGS and RNAseq data (see “Materials and methods” and Supplementary Table S3), lie notably near processing sites and might thus affect pre-rRNA processing through modification of RNA folding and RNA-RNA interactions, as described earlier^30,31^. Other variants in 5′ETS and ITS1 that can potentially influence human 18S rRNA maturation and the ribosome biogenesis are indicated in Fig. 5. Most of these variants were again independently validated using WGS and RNAseq data (Supplementary Table S4). A few of the variants are located in a *cis*-acting sequence that is highly conserved in mammals in Box C (Fig. 5 and Supplementary Fig.S10) with a possible function in ITS1 processing ^32^.

We also mapped the analyzed variants to well-known functional sites and annotated regions of rDNA units, including CTCF sites^33^; R repeat segments with Replication Fork Barrier Sites (RFBs), which are preferential sites of fork collapse in rDNA^34^, and an overlapping region corresponding to the 5′ region of a known rDNA lncRNA transcribed by pol II in an anti-sense direction. That ~ 12–16 kb transcript is called “promoter and pre-rRNA antisense” (PAPAS). PAPAS causes repression of rRNA synthesis through different pathways^27,35^. Our analysis showed that: (i) CTCF sites are highly conserved in functional rDNA units; only one variant was found at a CTCF site in a sequenced rDNA unit; and (ii) however, some (NN) variants described in the study overlapped the R repeat segments containing RFB sites (Supplementary Table S4). These variants are located in close proximity to the Sal-T1 and Sal-T2 boxes as well as inside of the box Sal-T3 (Supplementary Fig. S11) that can influence binding efficiency^36^. Downstream of box Sal-T5, a 20-nucleotide universal indel characteristic of both chromosomes 21 and 22 was found. Replication forks (RF) pause during normal replication at RFBs, where specific proteins impede the RF by binding tightly to DNA^34^. Thus, sequence variants in the region become candidates for functional interactions that may influence the complex replication of rDNA, which occurs in phases both early and quite late in S phase^37^.

## Discussion

Intra-genomic variation in rRNA gene copies is well documented in several species^38^; but in humans, the nucleolar organizer regions on the p-arms of acrocentric chromosomes contain rDNA arrays whose sequence and variability are only now being studied in detail. Subcloned rDNA fragments sequenced from several individuals found early evidence of variation—primarily single nucleotide variants (SNVs)—in the transcribed regions^8,39^. More recently, analysis of the whole-genome-sequencing data from the 1000 Genomes Project identified pervasive intra- and inter-individual nucleotide variation in the coding rDNA region^26^. However, none of the observed variants was validated in uncloned DNA, and there has been no comprehensive census of rDNA variants or their frequency within and between individuals. In our recent study, we presented data demonstrating an unexpectedly high level of rDNA variations within a single NOR region^20^. The average rate of variations in rDNA (roughly 7.5 variants per kb) is comparable to typical estimates of variations across the human genome.

The large number of rDNA units on chromosome 21 (~ 56 copies) made it very difficult to assemble a sequence of the entire NOR along with PJ and DJ regions in our previous work. Here we have focused on the NOR for chromosome 22, which typically carries a low number of rDNA repeats^3,12,13^, and isolated the entire block of rDNA units with their flanking PJ and DJ regions using a modified TAR cloning technique.

An entire rDNA array along with the flanking PJ and DJ regions in a single YAC/BAC molecule now provides a platform for assembly of a synthetic NOR region. This can enable detailed study of the sequence requirements and mechanism of nucleolar formation in human cells, including the role of PJ and DJ in this process. A Human Artificial Chromosome (HAC) vector containing multiple integrase sites for assembly of megabase-size genomic segments^40^ can be particularly useful. In human cells, a HAC with an indifferent DNA insert is maintained as a normal chromosome with no detectable co-localization with the nucleolus^40^. By contrast, when the well-characterized NOR is integrated into the HAC vector, the structural requirements for de novo formation and activity of a functional NOR can be investigated.

The fact that many alleles are evolutionarily conserved across ethnic groups and at levels near or at 50% is intriguing. It suggests that a variant may create a ribosome with an “alternative” conformation or functional property. Despite a couple of hot spots of variation (INDELs) in 28S rRNAs that located on the opposite strands of hairpin structures of ESs (see above), we see no global tendency for colocalization of pairs or groups of alleles in different repeat units. Thus, any effects might be intrinsic to individual alleles rather than based on cooperative action of multiple variants.

However, the null hypothesis is that such alleles arose during evolution and are indifferent in ribosome function, carried along as innocent bystanders. Additional studies can certainly clarify what sequences are required for various functions and whether variants affect their action. The positions and character of the variants suggest some possible effects.

It has long been known that alternative processing pathways all lead to the production of the mature ribosomal RNAs^30,41^, and it has been discussed that differential pathway use might be involved in a growing number of human disorders in which ribosome production is altered^32^. Among several universal ETS and ITS variants (i.e., found on both chromosomes 21 and 22 as well as in independent WGS and RNAseq data (see Supplementary Table S3), we found variants that lie near processing sites and might thus affect pre-rRNA processing through modification of RNA folding and RNA-RNA interactions^30,31,42,43^. Several variants in 5′ETS and ITS1 that can potentially influence human 18S rRNA maturation and ribosome biogenesis are highlighted in Fig. 5, Figure, Supplementary S10 and Supplementary Fig. S11. A few variants are located in a *cis* -acting sequence—C box that is highly conserved in mammals (Fig. 5 and Supplementary Fig. S10) with a possible role in ITS1 processing^42^.

Our analysis showed that some variants described in the study overlapped the R repeat segments with RFB sites (Supplementary Table S4). Sequence variants in this region are located in close proximity to the Sal-T1 and Sal-T2 boxes as well as within box Sal-T3 and in the vicinity of box Sal-T5, a 20-nucleotide universal indel that is characteristic for both chromosomes 21 and 22 (Supplementary Fig. S11), and become candidates for functional interactions that may influence rDNA replication.

The variations in mature rRNA sequence are suggestive. Both U682C in 18S and several variants in 28S rRNA ES are predicted to be riboSNitches, which could be related to modifications in flexibility and formation of 3D contacts as well as ribosome maturation. Even variants in ES that are apparently “silent” could affect vital functions like peptide bond formation. In this regard, the extensive microbial literature on ribosome function early on showed that variants in ribosome structure can affect miscoding, readthrough of nonsense mutations, and antibiotic resistance. For example, low levels of streptomycin increase miscoding; streptomycin resistant mutations decrease it; and streptomycin-dependent mutations slow ribosome movement [summarized in^44^]. In mammalian cells, work is more fragmentary, but it has been shown, for example, that macrolide antibiotics like sparsomycin “tighten” ribosomes to restrict the readthrough of frameshift sequences^45^—a process that is required, for example, for the production of a coronavirus protein^46^.

Ribosomes and nucleolar function have been further implicated in several types of pathology. Speculations about effects of variants on ribosome function, which are now open to direct test, have extended from alterations in the selection or efficiency of translation of particular mRNAs to consequences for the control of growth and the severity of “ribosomopathies” like Diamond-Blackfan anemia^47^.

## Materials and methods

### Hybrid cell lines

Four mouse/human monochromosomal hybrid cells containing the human chromosome 22 were used: A9HyTK-22^13^, A9#22(γ2)^16^, GM10888^17^, and GM13258^18^. The cells were cultured in Dulbecco’s modified Eagle’s medium supplemented with 10% fetal bovine serum (Atlanta Biologicals, Lawrenceville, GA, USA) and 400 μg/ml Hygromycin B for A9HyTK-22 and 800 μg/ml G418 (InvivoGen) for A9#22(γ2) at 37 °C in 5% CO_2_. The A9#22(γ2) cell line was obtained from Dr. Mitsuo Oshimura.

### Southern blot hybridization analysis

Genomic DNA was prepared in agarose plugs (0.5 × 10^6^ cells per plug) and restriction digested with EcoRV in the buffer recommended by the manufacturer. The digested DNA was separated by CHEF (CHEF Mapper, Bio-Rad) (autoprogram, 5–250 kb range, 16 h run), transferred to nylon membrane (Amersham Hybond-N+), and blot-hybridized with a 421-bp probe specific for the rDNA chromosome 22 spacer sequence. The DNA sequence for the probe was amplified by PCR using the primers FOR. 5′-GGGAGTCCGAGACAGAATGA-3′, REV. 5′-AGCTCCTGTGGTTTCAGGTG-3′. The blot was incubated for 2 h at 65 °C in pre-hybridization Church’s buffer (0.5 M Na-phosphate buffer containing 7% SDS and 100 µg/ml of salmon sperm DNA). The labeled probe was heat denatured in boiling water for 5 min and snap-cooled on ice. The probe was added to the hybridization buffer and allowed to hybridize overnight at 65 °C. The blot was then washed twice in 2 × SSC (300 mM NaCl, 30 mM sodium citrate, pH 7.0), 0.05% sodium dodecyl sulfate (SDS) for 10 min at room temperature, then twice in 2 × SSC, 0.05% SDS for 5 min at 60 °C, twice in 0.5 × SSC, 0.05% SDS for 5 min at 60 °C and twice in 0.25 × SSC, 0.05% SDS for 5 min at 60 °C. The blot was exposed to X-ray film for 24–72 h at − 80 °C to visualize labeled DNA band(s).

### Quantitative real-time PCR

Human rDNA copy number was estimated with a set of primers for intergenic spacer (IGS) (rDNA 122992, rDNA 129514, rDNA 134304 and rDNA 142197; sequences of all primers used in the study are in Supplementary Table S1) by quantitative real-time PCR using total human genomic DNA (Promega) and genomic DNA from mouse/human hybrid cell lines containing the human chromosome 22 in mouse background, i.e. A9HyTK-22, A9#22(γ2), GM10888, and GM13258. As an internal control for a single copy region, we used two pairs of primers specific for the human chromosome 22 PMM1 region.

### Construction of TAR vectors

Three TAR vectors, pJH6, pJH13 and pJH42, were constructed using the basic shuttle vector pJYB containing a YAC cassette (a yeast selectable marker HIS3 and a yeast centromere CEN6) and a BAC cassette [a bacterial chloramphenicol (CAM^R^) marker and an origin of replication oriF1]^20^ (Supplementary Fig. S1). The presence of the BAC cassette allows the TAR-cloned material to be directly transferred from yeast to *E. coli* cells, in which they propagate as bacterial artificial chromosomes, facilitating the purification of BAC DNA for sequencing. pJYB vector also includes the pUC vector containing the Amp^R^ marker and the pBR322 origin of replication that makes the pJYB vector multicopy. pJH42 vector contains two unique hooks, H1 and H4, that were PCR-amplified from the AC145212 BAC and KC876027 cosmid, respectively. pJH6 and pJH13 vectors contain two unique hooks that were PCR-amplified from KC876027 cosmid and AL592188 BAC, respectively. The hooks were cloned into the basic pJYB vector as SalI-AscI and NotI-XbaI fragments flanking the pUC linker (Supplementary Fig. S2). Before TAR cloning experiments, these TAR vectors were linearized with AscI/NotI digestion (these sites flank the pBR322 origin sequence and the Amp^R^ marker) to exposure of 5′ and 3′ targeting sequences. Sequences of the primers used in this study are presented in Supplementary Table S1.

In FISH and DNA combing experiments, JH50-BAC clone for DJ was used. JH50-BAC (46,161 bp) was sub-cloned from AL592188 BAC (161,802 bp) using TAR cloning (Supplementary Fig. S4). For this purpose, pJYB DJ TAR vector containing two unique hooks, 191 bp 5′hook and 170 bp 3′hook, was constructed. Before TAR cloning experiments, the TAR vector was linearized with AscI/NotI digestion to exposure of 5′ and 3′ targeting sequences. Sequences of the primers used for construction are presented in Supplementary Table S1.

### Preparation of genomic DNA

High molecular weight genomic DNA from A9HyTK-22 hybrid cells in agarose plugs was prepared as previously described^14,48^.

### Yeast strain and spheroplast transformation

For TAR cloning experiments, the highly transformable *Saccharomyces cerevisiae* strain VL6-48 (MATα, his3-Δ200, trp1-Δ1, ura3-52, lys2, ade2-101, met14), was used^14^. For yeast spheroplast transformation, 2–3 μg of high molecular weight genomic DNA isolated from the A9HyTK-22 cells was prepared in agarose plugs^14,48^, mixed with ~ 1 μg of the linearized TAR vector, and added to freshly prepared yeast spheroplasts. Yeast transformants were selected on synthetic complete medium plates lacking histidine (His^-^). The yield of His^+^ transformants per 2–3 μg of genomic DNA using ~ 1 μg of the TAR vector and 5 × 10^8^ spheroplasts varied from 30 to 100 colonies per plate.

To identify the clones harboring the desired region, the His^+^ transformants were combined into pools, each containing 24–30 transformants, and tested with the diagnostic primers specific for the targeted chromosome 22 regions. To find individual region-containing clones, His^+^ transformants from region-positive pools were tested with diagnostic primers listed in Supplementary Table S1.

### Physical characterization of TAR/YAC/BAC clones

Several steps were taken to characterize the TAR isolates. First, to prove the presence of the predicted genomic sequences in the TAR/YAC isolates, DNA from yeast His^+^ clones was examined by PCR using a set of pairs of the primers specific for each target region. Second, TAR isolates containing the entire target region were moved from yeast to bacterial *E.coli* cells by electroporation. In brief, yeast chromosome-size DNAs were prepared in agarose plugs and, after melting and agarase treatment, the DNAs were electroporated into DH10B competent cells (Gibco/BRL) using a Bio-Rad Gene Pulser with the setting 1.8 kV, 200 ohm, and 25 μF as previously described^14,48^. Third, to determine the size of the cloned inserts in the region-positive BAC clones, BAC DNAs were digested with endonucleases that cut only in the vector portion, separated by contour-clamped homogeneous electric field (CHEF) gel electrophoresis, and stained with EtBr. Finally, the predicted size of BACs was checked again by pairs of primers specific for each target region.

### Fluorescence in situ hybridization (FISH) analysis

FISH analysis was performed as previously described^49,50^. To obtain metaphase spreads, A9HyTK-22 and RPE cells were incubated in growth medium with 0.05 μg/ml of Colcemid (Gibco) overnight. Medium was aspirated and plates washed with 1 × PBS. Cells were removed from the plate by 0.25% Trypsin, washed off with DMEM, pelleted and resuspended in 10 ml of 50 mM KCl hypotonic solution for 30 min at 37 °C. Cells were fixed by three washes with fixative solution (75% acetic acid, 25% methanol). Between washes, cells were pelleted by centrifugation at 900 rpm for 4 min. Metaphase cells were evenly spread on a microscope slide and the fixative solution evaporated over boiling water and allowed to age 2 days at room temperature. Metaphase chromosomes on the slide were denatured in 70% formamide/2 × SSC for 2 min at 72 °C. Samples were dehydrated through a 70%, 90% and 100% ethanol series for 4 min each and left to air-dry. The probes were JH42-BAC for PJ, JH10-BAC for IGS and JH50-BAC for DJ. BAC DNAs were labeled with Green 496 dUTP (#42831, Enzo), Orange 552 dUTP (#42842, Enzo), or Red 650 dUTP (#42522, Enzo) using a nick translation DNA labeling system 2.0 (#GEN111-50, Enzo), respectively. 100 ng of each labeled probe was denatured in hybridization solution at 78 °C for 10 min and left at 37 °C for 30 min. The hybridization probe solution was applied to the sample and incubated at 37 °C overnight. Slides were washed with 0.4 × SSC, 0.3% Tween 20 for 2 min at 72 °C, briefly rinsed with 2 × SSC, 0.1% Tween 20, and air-dried in darkness. The samples were counterstained with VectaShield mounting medium with DAPI (Vector Labs). Slides were analyzed by fluorescence microscopy. Imaging was performed using DeltaVision microscopy in the CRC/LRBGE Fluorescence Imaging Facility (NIH).

### PacBio sequencing and assembly

BAC DNA extraction and sequencing were performed as in^20^. DNA from six TAR/BACs was isolated, mechanically sheared, and size selected. Separate libraries were constructed for each BAC and sequenced on a PacBio RSII instrument using one SMRTcell each and the P6-C4 chemistry. Raw sequencing reads were screened against the *E. coli* K12 reference genome to remove cloning contaminants prior to assembly. Screened reads were then assembled using Canu 1.7 using default parameters and genomeSize = 100 k^51^, and then polished using signal-level read information with Arrow SMRTtools 5.1.0^52^. Valid assemblies were expected to contain the vector sequence, a degree of circular overlap, and the hook sequences used for cloning. Vector sequence and circular overlaps were identified using MUMmer 3.23^53^ and removed, and the assemblies were re-oriented toward the first base remaining after the removal of vector sequence. The assemblies were then further examined a second time with Arrow to optimize accuracy.

### Analysis of variants and RNA structures

RNA secondary structures were predicted using methods based on global and local free energy estimation or minimum free energy consensus structure for aligned RNA sequences. Mature and pre-rRNAs were computationally folded containing alternative allelic variants and the minimum free energy of the corresponding secondary structure was calculated for different window sizes, as described in^54^.

Energy minimization was performed using the dynamic programming method and Afold algorithm for evaluation of internal loops^55^. The free-energy penalty associated with breaking (opening) of local secondary structure (target structure opening, Δ*G* kcal/mol) was estimated considering local disruption of secondary structure in windows of different length. Free-energy changes were approximated with nearest-neighbor free-energy parameters using the Afold program^55^, the Hybrid software^56^, and the OligoWalk program^57^. Local structure was considered for a set of suboptimal structures. Local free energy was estimated for pairs of highly similar sequences, extracted from pairwise alignments with length windows of 100 and 350 nucleotides^22^. The structural index (*D*) was defined as the Euclidean distance of base pairs.

Monte Carlo simulation and analysis of randomized sequences^58^ was used to estimate significant differences between target structure opening (*ΔG*) or structural index (*D*) of allelic variants. The free-energy penalty associated with local secondary structure opening (Δ*G* kcal/mol) or structural index (*D*) for all random sequences was calculated for local disruption of secondary structure in a given window. *P*-values for randomizations and for difference between variant alleles were determined by the MWW test.

The RNAalifold program was used to predict RNA consensus structures based on multiple sequence alignments^59^ (Vienna package—http://rna.tbi.univie.ac.at). Base-pair distance was computed as the average distance of two single sequences, and a consensus sequence derived by RNAalifold. *z*-scores and empirical *P*-values was also estimated, based on a base-pair distance measure. One hundred random samples were used to estimate empirical *P*-values. RNAsnp software^60^ was applied with the variants to detect local RNA secondary structure changes.

## Supplementary Information

Supplementary Information 1.

Supplementary Table S4.

## Data Availability

Supplementary Table S2A and B list all sources and accession numbers.
