# Supplementary material for: The genomic structure of a human chromosome 22 nucleolar organizer region determined by TAR cloning
Source: Sci Rep. 2021 Feb 4;11:2997. doi: 10.1038/s41598-021-82565-x (PMC7862453; doi:10.1038/s41598-021-82565-x)
Supplement: Supplementary file 1 — Supplementary Information 1. [file 41598_2021_82565_MOESM1_ESM.docx]

**Supplementary Data**

**The genomic structure of a human chromosome 22 nucleolar organizer region determined by TAR cloning**

Jung-Hyun Kim^1#^, Vladimir N. Noskov^1#^, Aleksey Y. Ogurtsov^2#^, Ramaiah Nagaraja^3^, Nikolai Petrov^1^, Mikhail Liskovykh^1^, Brian P. Walenz^4^, Hee-Sheung Lee^1^, Natalay Kouprina^1^, Adam M. Phillippy^4^, Svetlana A. Shabalina^2^*, David Schlessinger^3^* and Vladimir Larionov^1^*

^1^National Cancer Institute, Developmental Therapeutics Branch, Bethesda, MD 20892, USA

^2^National Center for Biotechnology Information, National Library of Medicine, Bethesda, MD 20892, USA

^3^National Institute on Aging, Laboratory of Genetics and Genomics, Baltimore, MD 21224, USA

^4^National Human Genome Research Institute, Computational and Statistical Genomics Branch, Bethesda, MD 20892, USA





**Supplementary Fig. S1**

**Sequencing of DNA isolated from EcoRV and ApaLI fragments by Illumina. (A)** Southern blot analysis of the A9HyTk-22 hybrid cell line. **(B)** Based on the southern blot analysis, rDNA repeats containing gels were cut out from the CHEF gel after EcoRV or ApaLI endonuclease digestion. M – CHEF DNA Size Lambda Ladder (BIO-RAD) **(C)** After agarose gel digestion, isolated DNAs were amplified using a single cell DNA amplification method. **(D)** Amplified DNAs were sequenced and analyzed by illumina next-generation sequencing. **(E)** This panel shows illumina sequencing reads to PJ cosmid (KC876027) / DJ BAC (AL592188) on 5 kb upstream and 5 kb downstream of the innermost EcoRV or ApaL1 enzyme recognition site (gel sequencing reads each end) in the A9HyTk-22 hybrid cell line. Designing of pJH13 EcoRV or pJH6 ApaL1 TAR cloning vectors based on illumina sequencing reads.


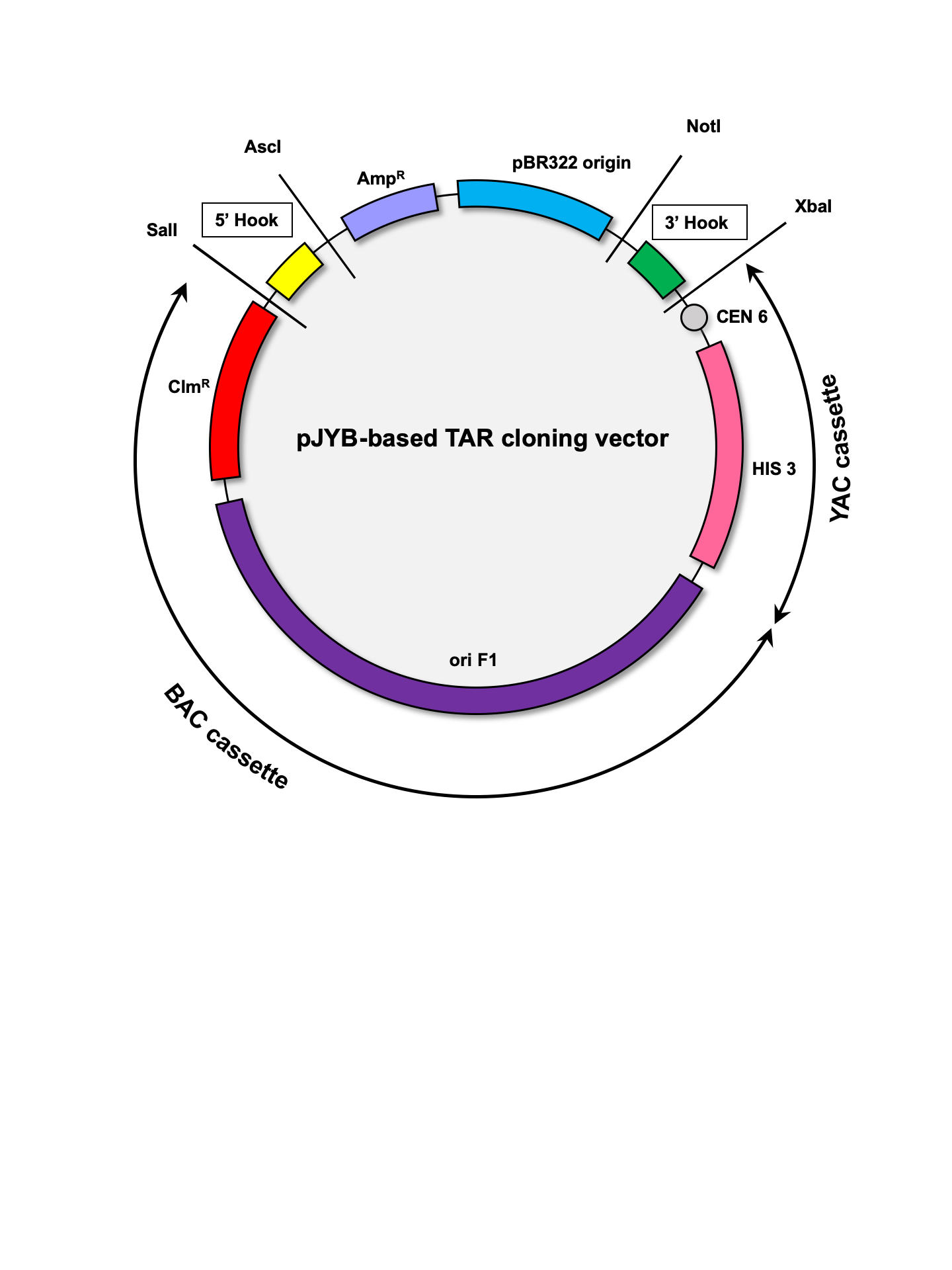


**Supplementary Fig. S2**

**Scheme of the basic shuttle vector pJYB.** The vector contains a YAC cassette (a yeast selectable marker HIS3 and a yeast centromere CEN6) and a BAC cassette [a bacterial chloramphenicol (Clm^R^) marker and an origin of replication oriF1]. Thus, the vector may propagate in yeast as well as in bacterial *E. coli* cells. 5’ Hook and 3’ Hook are inserted into the vector as SalI/AscI and NotI/XbaI targeting sequences, correspondingly. The pUC linker containing the Amp^R^ marker and the pBR322 origin of replication makes the pJYB vector multicopy. Before TAR cloning experiments, pJYB vector is linearized by AscI/NotI to make the hooks highly recombinogenic.


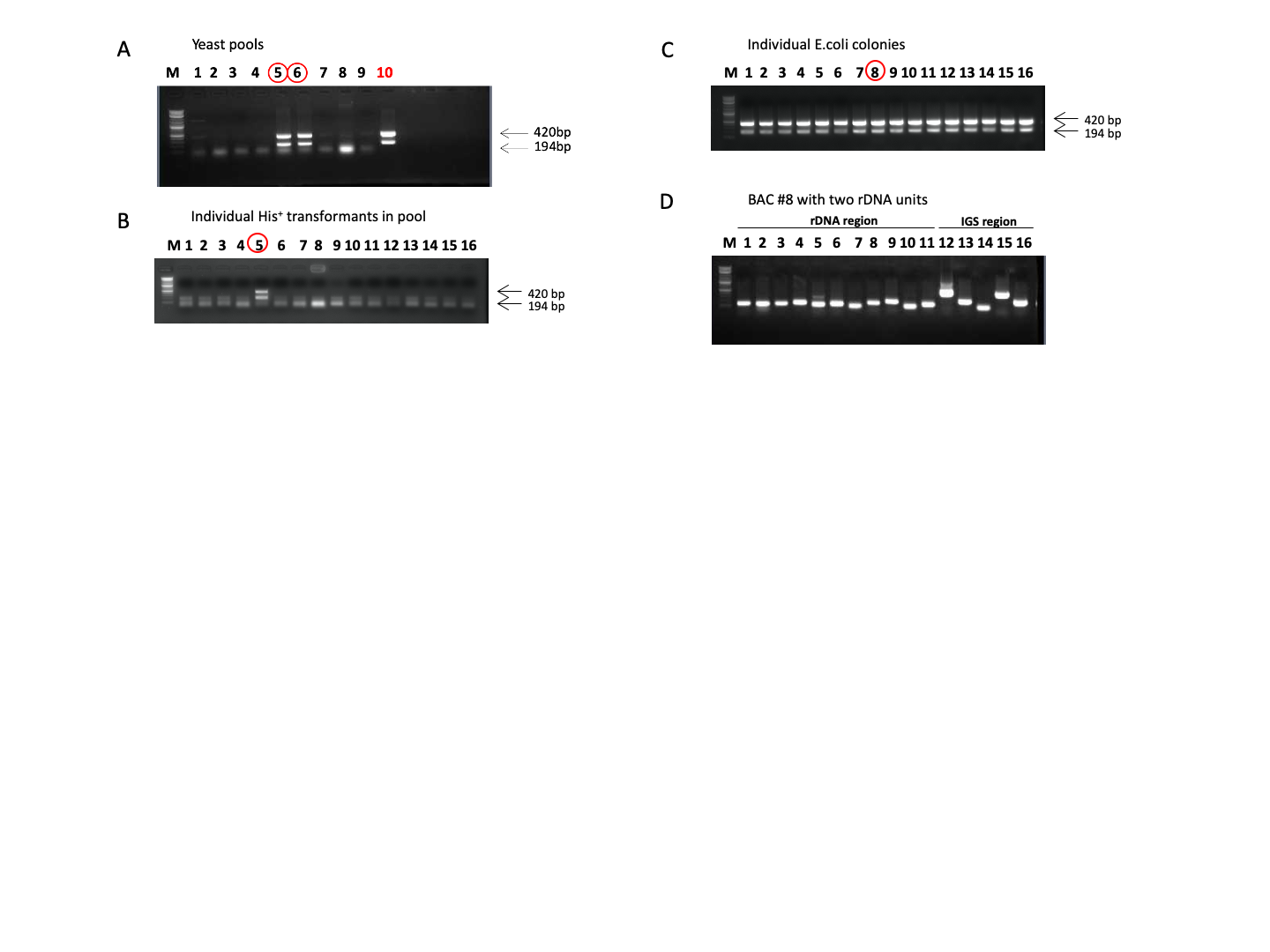


**Supplementary Fig. S3**

**Analysis of yeast and bacterial clones obtained with pJH6 vector. (A)** The pools were screened by two pairs of diagnostic primers, i.e. 14F/14R (region 14 in IGS) and 16F/16R (region 16 in IGS) (Supplementary Table S1). Pools #5, #6 and #10 are regions-of interest-positive. Size of the PCR products are 420-bp and 194-bp, correspondingly. **(B)** As an example, PCR analysis of 16 individual His^+^ transformants obtained from a region of interest-positive pool by two pairs of diagnostic primers, i.e. 14F/14R (region 14 in IGS) and 16F/16R (region 16 in IGS) (Supplementary Table S1). Size of the PCR products are 420-bp and 194-bp, correspondingly. Transformant #5 is positive. **(C)** PCR analysis of Clm^R^ bacterial colonies obtained from the yeast region-positive clone #5 by two pairs of diagnostic primers, i.e. 14F/14R (region 14 in IGS) and 16F/16R (region 16 in IGS) (Supplementary Table S1). Size of the PCR products are 420-bp and 194-bp, correspondingly. All clones are positive. **(D)** PCR analysis of DNA from BAC#8 with a set of 16 pairs of diagnostic primers for 18S, 5.8S, 28S and IGS. M - Quick-Load 1 kb Plus DNA Ladder (New England BioLabs).

**Supplementary Fig. S4**

Schematic diagram shows a TAR cloning procedure of JH50/DJ clone for distal junction (DJ) used as a FISH and DNA combing probe. The JH50/DJ-BAC was sub-cloned from AL592188 BAC (161,802 bp) to make a specific probe for the distal junction region along with a part of rDNA intergenic spacer (IGS) to see an orientation of the rDNA repeats. JH50/DJ TAR isolate was transferred from yeast to *E. coli* cells for BAC DNA isolation. The length of the insert in the JH50/DJ-BAC is 46,161 bp. Sequences of the primers used for the TAR vector construction are listed in Supplementary Table S1.

**
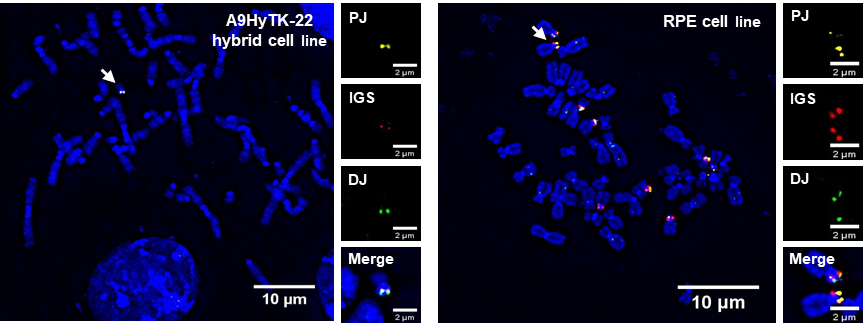
**

**Supplementary Fig. S5**

FISH analysis of the rDNA cluster with the flanking PJ and DJ regions in A9HyTK-22 hybrid cell line containing chromosome 22 and human RPE cells using three specific probes: JH42-BAC for PJ (yellow), JH50-BAC for DJ (green) and JH10-BAC for IGS (red). Color-bars show the signal distributions on the acrocentric chromosomes.


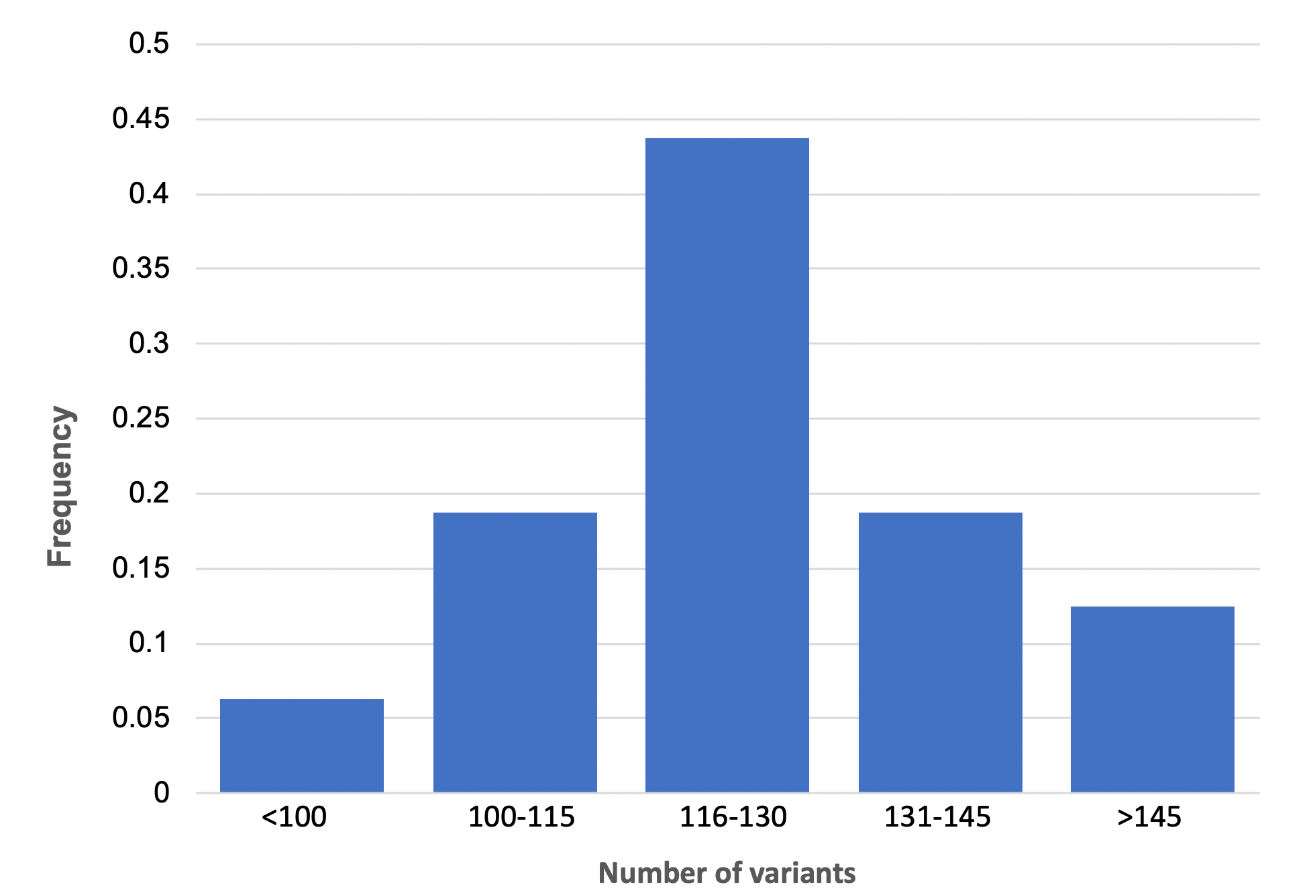


**Supplementary Fig. S6**

Distribution of the number of variants in pairwise comparisons between complete rDNA units in chromosome 21 and 22 (intra- & inter- individual pairwise comparisons of complete rDNA units from chromosomes 22 and 21 with their suitable reference sequence from our previous publication ^20^ (GenBank accession [KY962518](https://www.ncbi.nlm.nih.gov/nuccore/KY962518)).

**
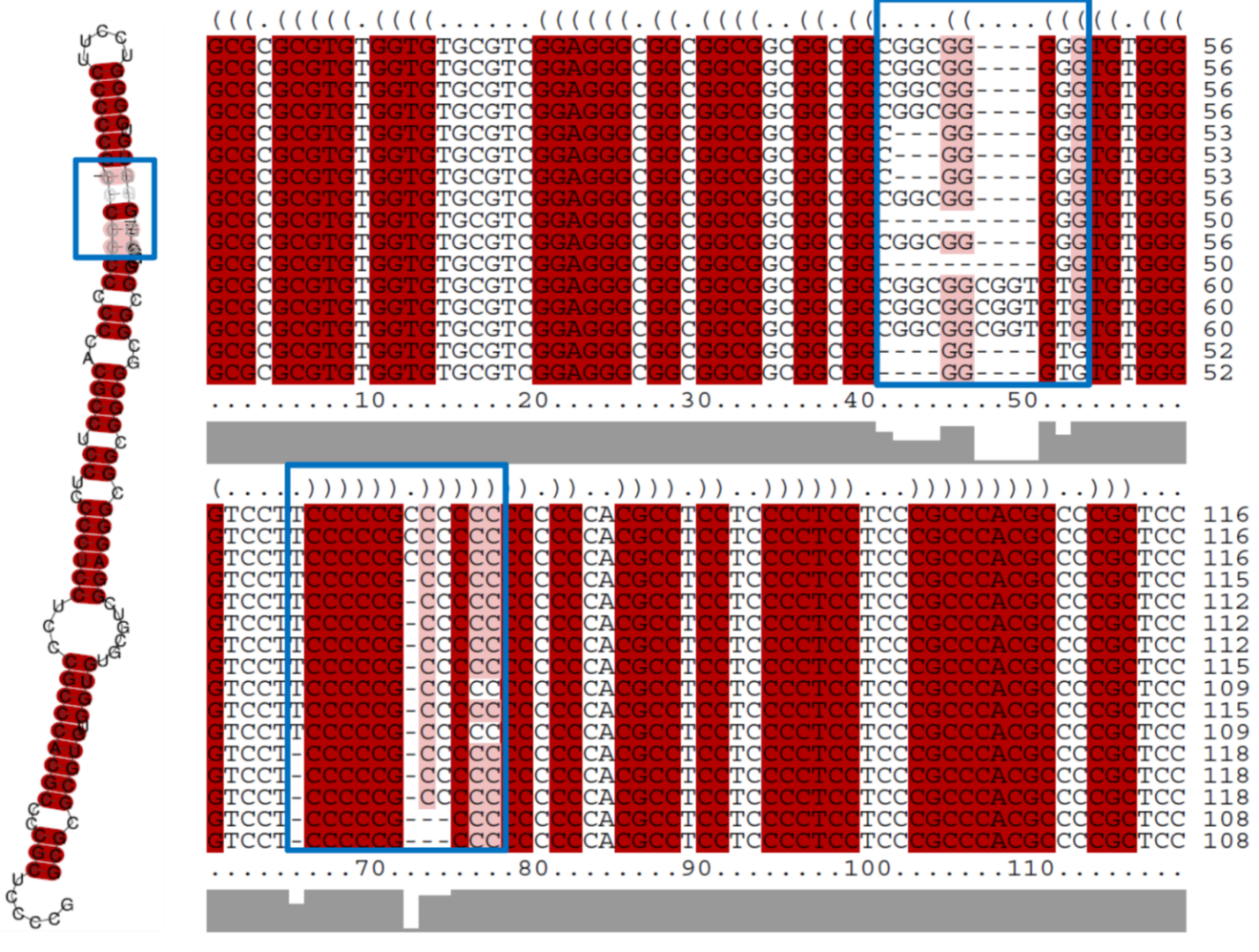
**

**Supplementary Fig. S7**

**Multiple alignment (right panel) and consensus folding (hairpin structure - left panel) of ES15L variants.** Variants positions are shown in light red colors. Two hot spots of variability are located on opposite strands of the same stem-loop structure (shown in blue boxes).

**Supplementary Fig. S8**

**Consensus folding of the second hairpin from ES27L region.** Two hot spots of variability are located on the opposite strands of the same stem-loop structure (sequences are shown in blue boxes; 2D rRNA structure - modified from Kim et al. ^20^).

**
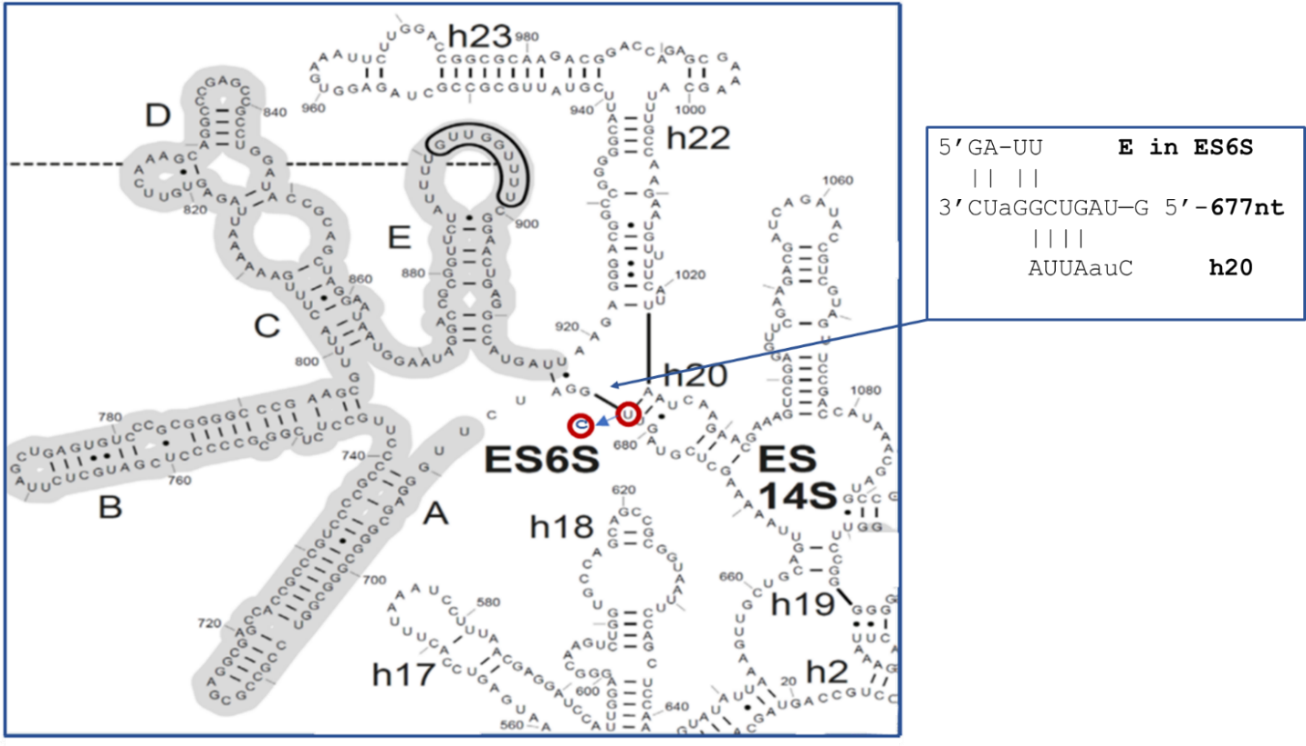
A**

**B**

**
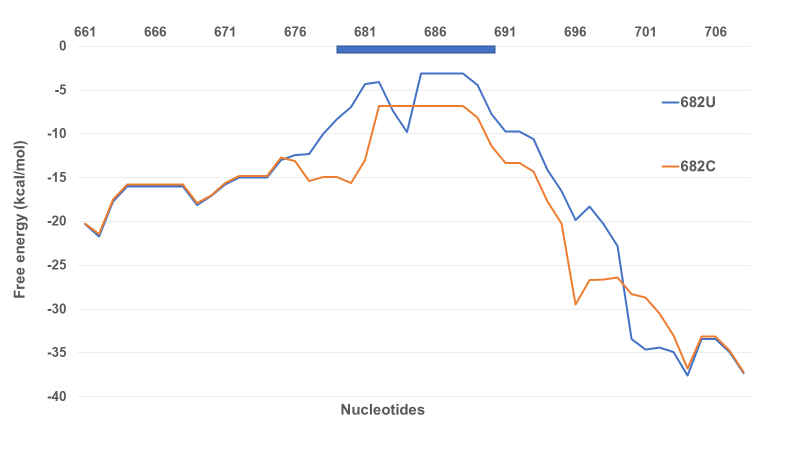
**

**Supplementary Fig. S9**

Secondary structure of 18S rRNA in the immediate vicinity of ES6S and ES14S. The predicted RiboSNitch at position U682C and the modified pairing patterns are shown in blue box. This variant is located at a known RNA-RNA methylation guide interaction site mapped at positions 679-688, with methylation at position 684 in the human 18S rRNA. Such RNA-RNA interactions have key functions in the modification, processing and dynamic folding of rRNA ^1,2^. **(B)** Free energy profiles (ΔG of target opening is estimated as described in Materials and Methods for 18S rRNA RiboSNitch at position U682C. RNA-RNA methylation guide interaction site mapped at positions 679-688 as blue box, with methylation at position 684 in the human 18S rRNA (blue star).

**References**

1. Matveeva, O. V. & Shabalina, S. A. Intermolecular mRNA-rRNA hybridization and the distribution of potential interaction regions in murine 18S rRNA. Nucleic Acids Res 21, 1007-1011, doi:10.1093/nar/21.4.1007 (1993).

2. Watkins, N. J. & Bohnsack, M. T. The box C/D and H/ACA snoRNPs: key players in the modification, processing and the dynamic folding of ribosomal RNA. Wiley Interdiscip Rev RNA 3, 397-414, doi:10.1002/wrna.117 (2012).

**A** **B**

**
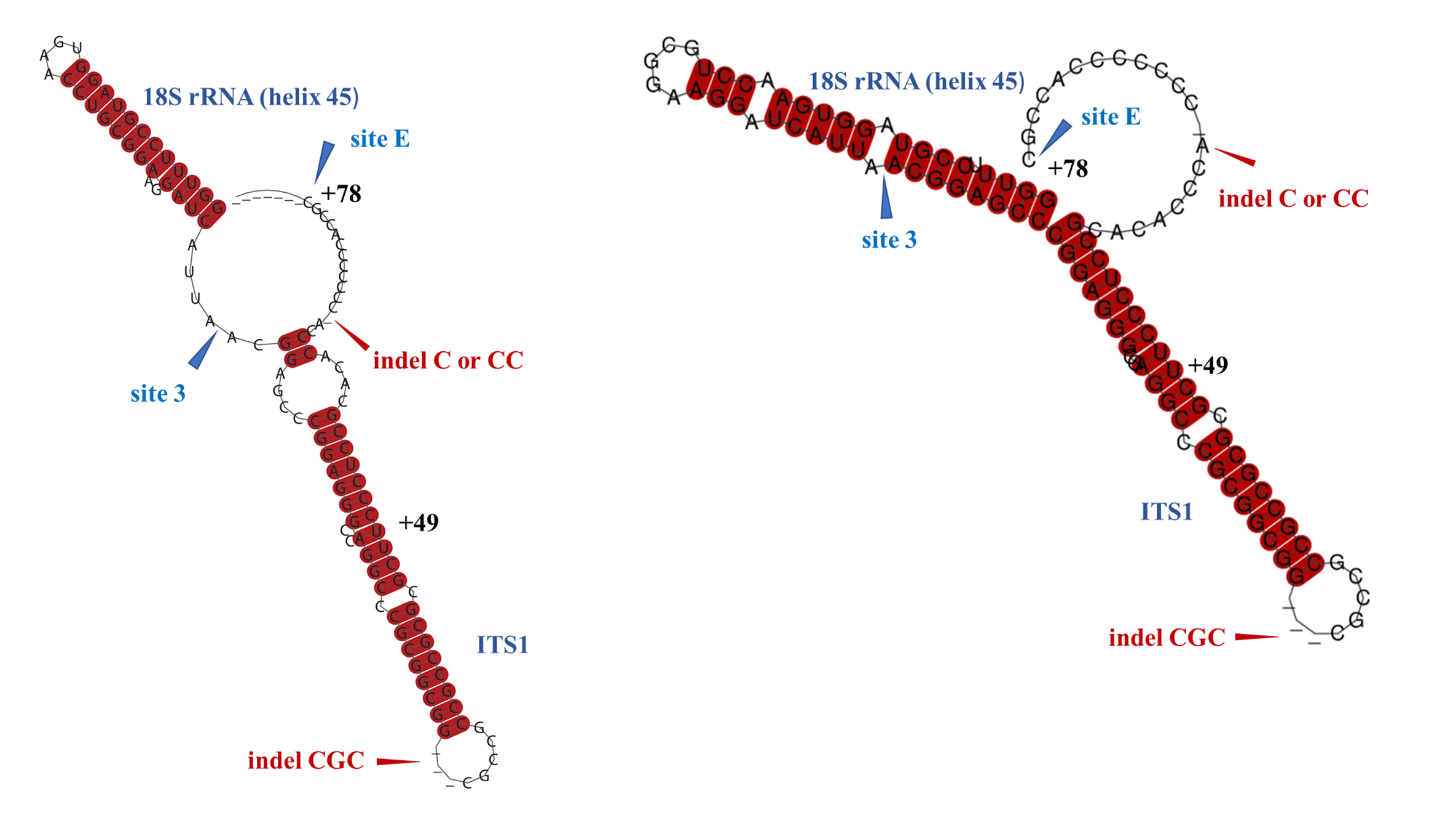
**

**Supplementary Fig. S10**

(**A**) Predicted RNA secondary structures of the 3’ end of mature rRNA and ITS1 (using the Alifold program). This region includes the helix 45 of 18S rRNA, followed by the ITS1 sequence up to the distal position of the processing site E (nt +78 relative to the 3’ end of mature 18S rRNA). The nucleotide lengths are shown in black numbers, and nucleotides labeled “+” sign show the length of the ITS1 sequence as indicated in 47S rRNA. (**B**) Predicted alternative consensus RNA secondary structures of the 3’ end of mature rRNA and ITS1. The 18S rRNA and ITS1 sequences are folded together; there is no classical helix 45 of 18S rRNA. The consensus structure and pairing probability showed that the GCG indel could participate in the modulation of the optimal and sub-optimal structures in the region as well as in changing the loop size in the ITS1 hairpin.

**Supplementary Fig. S11**

The location of variants in the immediate vicinity of the Sal-boxes T1 – T5 and TTF-I binding sites in human 47S rDNA. Sal-boxes are shown in green; variants are marked in yellow.

**Supplementary Table S1**

List of primers used in this study

|  | **Primer Name** | **Forward (5’ to 3’)** | **Reverse (5’ to 3’)** |
| --- | --- | --- | --- |
|  | **rDNA Diagnostic primers** | | |
| 18S | rDNA 105734 (region 1) | TGTCAGGCGTTCTCGTCTC | GACGTCACCACATCGATCAC |
|  | rDNA 106285 (region 2) | GAACGGTGGTGTGTCGTTC | GCGTCTCGTCTCGTCTCAC |
|  | rDNA 107548 (region 3) | CGTGCCTGAGGTTTCTTC | GGGGGAAGAAGAGGATCG |
|  | rDNA 109524 (region 4) | AAACGGCTACCACATCCAAG | CCTCCAATGGATCCTCGTTA |
|  | rDNA 109952 | ACCGCGGTTCTATTTTGTTG | GACAAATCGCTCCACCAACT |
| 5.8S | rDNA 112056 (region 5) | GTCGATGAAGAACGCAGCTA | GACGCTCAGACAGGCGTAG |
| 28S | rDNA 113431 (region 6) | AGTAACGGCGAGTGAACAGG | GCCTCGATCAGAAGGACTTG |
|  | rDNA 113678 (region 7) | GCTAAATACCGGCACGAGAC | TTCACGCCCTCTTGAACTCT |
|  | rDNA 114860 | TTAGGACCCGAAAGATGGTG | CCTTTTCTGGGGTCTGATGA |
|  | rDNA 115727 (region 8) | GAACTTTGAAGGCCGAAGTG | ATCTGAACCCGACTCCCTTT |
|  | rDNA 116970 | CAGGGGAATCCGACTGTTTA | ATGACGAGGCATTTGGCTAC |
|  | rDNA 117701 (region 9) | TGGGTTTTAAGCAGGAGGTG | GACGGTCTAAACCCAGCTCA |
|  | rDNA 118316 (region 10) | CCATTCGTAGACGACCTGCT | CTTGTGTCGAGGGCTGACTT |
| IGS  (spacer) | rDNA 119957 (region 11) | CGGAACTCCCTCTCCTACATT | AAGAAAACGCAAGGCAAAA |
|  | rDNA 122992 | ACACAGATGAGACGCACGAG | GAGAGAGACCAATCCCACCA |
|  | rDNA 124231 | GCCCACACCGTTTTTAATTG | TACGAAATCAGCCAGACGTG |
|  | rDNA 125032 | ATACCTCAGCCGACACATCC | CCTCCTCAAACGCAAGAAAG |
|  | rDNA 125132 | CCTTTCTTGCGTTTGAGGAG | CAGGTCAAGGAGGTGGTGTT |
|  | rDNA 125632 | AACGAAACACCACCTCCTTG | AAAGAACGGGCCCAAATACT |
|  | rDNA 126263 (region 12) | GTGTTCCCGTGAGGAGTGAT | ACAGAGAGAAGGCCCTAGCC |
|  | rDNA 126708 | GGCTAGGGCCTTCTCTCTGT | GCTGAACGTTCTCCCAAAAG |
|  | rDNA 128855 | TTCTCTGGCTGACTGCCTGT | CGACACAGTGAGAGAGAAAGG |
|  | rDNA 129514 | TATGATTTCGGGAGGTCGAG | CCCTTAGGACGCCGTTTAT |
|  | rDNA 134304 (region 13) | CACCTAGCGGCCACTGTTAT | AGTAAAGCGCCGATCAAAGA |
|  | rDNA 135055 | GGCTCTAGTCTGGGCCTTCT | AACGGCAGCTAACGTGTCTT |
|  | rDNA 137127 | ATCTGCCCGTGTCCTAAGTG | CTGGGCAGGAAAGTTCTCAG |
|  | rDNA 138700 (region 14) | GAATGCGACTCCTGCAAAAT | TTCCCAAGTCTGGTTGATCC |
|  | rDNA 140098 | AGTACCGGCACCATCCATAG | TTAGGGCGTGAGCTTGTCTT |
|  | rDNA 141057 | CCCTCTGCGAGAAGACAGAC | GGAGACGGACCCTATTGACA |
|  | rDNA 141247 | ACAGCTGCTGGTCCTCCTAA | TTGCAGAAGGGGATCAAATC |
|  | rDNA 141499 | GGACGAGGTCCAAGTGTTGT | TTGCAGAAGGGGATCAAATC |
|  | rDNA 142197 (region 15) | GGGAGTCCGAGACAGAATGA | TGTGCAGACCGAATCAGAAG |
|  | rDNA 142418 | CGCTGACTCCCTCTACCTTG | AGCTCCTGTGGTTTCAGGTG |
|  | rDNA 144016 (region 16) | AGGAGTCCCCTGGTCTGTCT | GTCAAGGTCCAAACCGAAAA |
|  | rDNA 144469 | CTCACAGAGGAAGGGAGCAC | GGTCAATCAGACCCAGTCGT |
|  | rDNA 148165 | GGAAGAGCCTACGCATTCTG | CGCGACAGAGTCAGAAGACA |
|  | rDNA 150089 | CCTCCAGTGGTTGTCGACTT | GAACGACACACCACCGTTC |
|  | rDNA 154279 | CCTGCGGCTTAATTTGACTC | GACAAATCGCTCCACCAACT |
|  | **Primers for construction of targeting sequences (hooks) in TAR vectors** | | |
| pJH42  pJH6  pJH13 | Hook 1 - H1 (166 bp)  Hook 2 – H4 (116 bp) | GTGGTAGCCGTTTCTCAGGCT  ACTTTCGTCTGTGGATGACCCA | TCTAGATAACCTCGGGCCGAT  AGCGCCGGAGCAGGTGCA |
|  | Hook 1 – ApaLI left (177 bp)  Hook 2 – ApaLI right (181 bp) | ATGGATAGCGAATTAAAATTAGATGTG  TTGCCTTCAAGTCCAGCGTC | TCATTCTCTCTGTTTCTGCCTC  GGCTGGAATGCAGTGGTGCA |
|  | Hook 1 – EcoRV left (196 bp)  Hook 2 – EcoRV right (199 bp) | TGTGGGAAGTTGACTCCAGCT  TTACAATTTGCTGAGGAGTGCTTT | ACTCCTGTGTCTCTTTCAGCTA  ATTAGACATATCAATGAGACAGATAG |
| pJH50 | Hook 1 – 5’H (170 bp)  Hook2 – 3’H (191 bp) | GAAGGTGGTGTCGGTGAGAT TCGCTTCTCCCCCCCAACCCC | AAGTATGCGATGCACCCTCT TCGGTGAGAAAAGCCTTCTCTAGCGA |
|  | **Human DNA copy number control primers** | | |
|  | Human Chr 22 PMM1 p2 | TGAGCAATGGTTGTTCTCCA | GTGGCTTCTCCAATGCAAAT |
|  | Human Chr 22 PMM1 p4 | TAGAGTGCAGATCGGTGTGG | AAGACGCCCAGGAATCTCTC |
|  | **Sequences of primers for junction between the vector part and insert** | | |
|  | PUC-diaF/ PUC-diaR | GACAAGGTGCTGATGCCGCT | GCTCCGGGAGACCGGCG |

**Supplementary Table S2**

GenBank accession numbers of the BACs and the contig in this study

**A**

| **Sequence name** | **GenBank accession number** |
| --- | --- |
| JH6/EcoRV-BAC | MT497459 |
| JH13/ApaLI-BAC | MT497387 |
| JH42-BAC | MT497460 |
| Chromosome22 NOR Region  from cell line A9HyTK-22 | MT497461 |

**B**

| **Sample** | **Type** | **Database** | **Accession / Link** |
| --- | --- | --- | --- |
| BACs | Illumina / PacBio / Nanopore / Assemblies | NCBI | https://www.ncbi.nlm.nih.gov/bioproject/?term=PRJNA380105 |
| NA12878 | *Illumina WGS* | EBI | https://www.ebi.ac.uk/ena/data/view/SAMEA1573618 |
| NA12878 | PacBio WGS | GIAB | ftp://ftp-trace.ncbi.nlm.nih.gov/giab/ftp/data/NA12878/NA12878_  PacBio_MtSinai/sorted_final_merged.bam |
| NA12878 | Nanopore WGS | AWS | https://github.com/nanopore-wgs-consortium/NA12878 |
| HX1 | PacBio WGS / Illumina WGS | NCBI | https://www.ncbi.nlm.nih.gov/bioproject/?term=PRJNA301527 |
| AK1 | PacBio WGS / Illumina WGS / Illumina RNA-Seq | NCBI | https://www.ncbi.nlm.nih.gov/bioproject/?term=PRJNA298944 |
| CHM1 | PacBio WGS / Illumina WGS | NCBI | https://www.ncbi.nlm.nih.gov/bioproject/?term=PRJNA246220 |
| K562 Short | Illumina RNA-Seq | ENCODE | https://www.encodeproject.org/experiments/ENCSR000CRB/ |
| K562 Long | Illumina RNA-Seq | ENCODE | https://www.encodeproject.org/experiments/ENCSR000CQA/ |

**Supplementary Table S3**

Sequence variants identified in unit 1 and unit 2 of rDNA on chromosome 22

|  |  | chr22_unit1 | |  | chr22_unit2 | |  |
| --- | --- | --- | --- | --- | --- | --- | --- |
|  |  | # variants | # deletions | freq | # variants | # deletions | freq |
|  | 5'ETS | 11 | 4 | 0.0827 | 8 | 1 | 0.0708 |
|  | 18S | 1 | 0 | 0.0075 | 0 | 0 | 0 |
|  | ITS1 | 7 | 4 | 0.0526 | 4 | 1 | 0.0354 |
|  | ITS2 | 5 | 2 | 0.0376 | 4 | 2 | 0.0354 |
|  | 28S | 13 | 8 | 0.0977 | 17 | 8 | 0.1504 |
|  | 3'ETS | 2 | 2 | 0.015 | 1 | 1 | 0.0088 |
|  | IGS-NT | 94 | 12 | 0.7068 | 79 | 20 | 0.6991 |
|  |  |  |  |  |  |  |  |
|  | Total rRNAs | 39 | 20 |  | 34 | 13 |  |
|  | Total unit | 133 | 32 |  | 113 | 33 |  |

**Supplementary Table S4**

Comparison of variants located on chromosomes 22 and 21 (available as a separate excel file). Positions of the chromosome 22 variants that match the location of the chromosome 21 variants are shown in column D (yellow - completely matching and red - partially overlapping). The positions of chromosome 22 variants that overlap the location of CTCF sites (shown in gray); and Replication Fork Barrier Sites -RFBs (shown in green) are highlighted in columns A and B. The positions of the chr22 variants that match the location of the chr21 variants are shown in column D (yellow - completely matching and red - partially overlapping).

The positions of chr22 variants that overlap the location of CTCF sites (shown in gray) and Replication Fork Barrier Sites -RFBs (shown in green) are highlighted in columns A and B.

**Supplementary Methods**

**Whole-genome validation and 45S expression analysis**. For whole-genome validation experiments we used four samples for which both high-coverage Illumina and PacBio data were available: AK1 (Korean ancestry), HX1 (Chinese ancestry), NA12878 (Caucasian ancestry) and CHM1 (Caucasian ancestry) ^1-6^. Illumina reads from the four validation samples were mapped to the modified reference genome, separately for each validation sample. To count as ‘validated’, an allele required at least 20 supporting read alleles in at least one sample. Variants in both the spacer and transcribed region were assessed in an analogous manner, employing a multiple and a pairwise sequence alignment containing the new reference sequence and sequences homologous to the new reference sequence extracted from the BAC.

Nucleolar RNA-seq sequencing data of the cell line K562 was obtained from ENCODE (see Table S2B), following Zentner et al. ^7^. The data include both ‘long RNA’ (2 × 76 bp Illumina reads) and ‘short RNA’ (1 × 36 bp Illumina reads) datasets generated by the Gingeras’s lab ^8^. RNA-seq data were also available for the AK1 sample (2 × 100 bp Illumina). Analogous to the steps described for the validation of 45S variants from DNA, the extracted sample alleles were projected onto the multiple sequence alignment of BAC-derived sequences. Only alleles with source column total coverage ≥100 were evaluated, and variants with >10% relative allele frequency were counted as ‘validated’.

**References**

1 Seo, J.S., Rhie, A., Kim, J., Lee, S., Sohn, M.H., Kim, C.U., Hastie, A., Cao, H., Yun, J.Y., Kim, J., Kuk, J., Park, G.H., Kim, J., Ryu, H., Kim, J., Roh, M., Baek, J., Hunkapiller, M.W., Korlach, J., Shin, J.Y., Kim, C. De novo assembly and phasing of a Korean human genome. Nature 538(7624), 243-247 (2006).

2 Thi, L., Guo, Y., Dong, C., Huddleston, J., Yang, H., Han, X., Fu, A., Li, Q., Li, N., Gong, S., Lintner, K.E., Ding, Q., Wang, Z., Hu, J., Wang, D., Wang, F., Wang, L., Lyon, G.J., Guan, Y., Shen, Y., Evgrafov, O.V., Knowles, J.A., Thibaud-Nissen, F., Schneider, V., Yu, C.Y., Zhou, L., Eichler, E.E., So, K.F., Wang, K. Long-read sequencing and de novo assembly of a Chinese genome. Nat Commun 7, 12065 (2016).

3 Steinberg, K.M., Schneider, V.A., Graves-Lindsay, T.A., Fultonm R.S., Agarwala, R., Huddleston, J., Shiryev, SA., Morgulis, A., Surti, U., Warren, W.C., Church, D.M., Eichler, E.E., Wilson, R.K. Single haplotype assembly of the human genome from a hydatidiform mole. Genome Res 24(12), 2066-2076 (2014).

4 Eberle, M.A., Fritzilas, E., Krusche, P., Källberg, M., Moore, B.L., Bekritsky, M.A., Iqbal, Z., Chuang, H.Y., Humphray, S.J., Halpern, A.L., Kruglyak, S., Margulies, E.H., McVean, G., Bentley, D.R. A reference data set of 5.4 million phased human variants validated by genetic inheritance from sequencing a three-generation 17-member pedigree. Genome Res 27(1), 157-164 (2017).

5 Pendleton, M., Sebra, R., Pang, A.W., Ummat, A., Franzen, O., Rausch, T., Stütz, A.M., Stedman, W., Anantharaman, T., Hastie, A., Dai, H., Fritz, M.H., Cao, H., Cohain, A., Deikus, G., Durrett, R.E., Blanchard, S.C., Altman, R., Chin, C.S., Guo, Y., Paxinos, E.E., Korbel, J.O., Darnell, R.B., McCombie, W.R., Kwok, P.Y., Mason, C.E., Schadt, E.E., Bashir, A. Assembly and diploid architecture of an individual human genome via single-molecule technologies. Nat Methods 12(8), 780-786 (2015).

6 Chaisson, M.J., Huddleston, J., Dennis, M.Y., Sudmant, P.H., Malig, M., Hormozdiari, F., Antonacci, F., Surti, U., Sandstrom, R., Boitano, M., Landolin, J.M., Stamatoyannopoulos, J.A., Hunkapiller, M.W., Korlach, J., Eichler, E.E. Resolving the complexity of the human genome using single-molecule sequencing. Nature 517(7536), 608-611 (2015).

7 Zentner, G.E., Saiakhova, A., Manaenkov, P., Adams, M.D., Scacheri, P.C. Integrative genomic analysis of human ribosomal DNA. Nucleic Acids Res 39(12):4949-4960 (2011).

8 Dobin, A., Davis, C.A., Schlesinger, F., Drenkow, J., Zaleski, C., Jha, S., Batut, P., Chaisson, M., Gingeras, T.R.mSTAR: ultrafast universal RNA-seq aligner. Bioinformatics 29(1):15-21 (2013).
